# Tricin levels and expression of flavonoid biosynthetic genes in developing grains of purple and brown pericarp rice

**DOI:** 10.7717/peerj.6477

**Published:** 2019-02-18

**Authors:** Alexander Poulev, Joseph R. Heckman, Ilya Raskin, Faith C. Belanger

**Affiliations:** Department of Plant Biology, Rutgers, The State University of New Jersey, New Brunswick, NJ, USA

**Keywords:** Tricin, Rice bran, Flavonoids

## Abstract

The methylated flavone tricin has been associated with numerous health benefits, including reductions in intestinal and colon cancers in animal models. Tricin is found in a wide range of plant species and in many different tissues. However, whole cereal grains, such as rice, barley, oats, and wheat, are the only food sources of tricin, which is located in the bran portion of the grain. Variation in tricin levels was found in bran from rice genotypes with light brown, brown, red, and purple pericarp color, with the purple pericarp genotypes having the highest levels of tricin. Here, we analyzed tricin and tricin derivative levels in developing pericarp and embryo samples of a purple pericarp genotype, IAC600, that had high tricin and tricin derivative levels in the bran, and a light brown pericarp genotype, Cocodrie, that had no detectable tricin or tricin derivatives in the bran. Tricin and tricin derivatives were detected in both the pericarp and embryo of IAC600 but only in the embryo of Cocodrie. The purple pericarp rice had higher total levels of free tricin plus tricin derivatives than the light brown pericarp rice. When expressed on a per grain basis, most of the tricin component of IAC600 was in the pericarp. In contrast, Cocodrie had no detectable tricin in the pericarp samples but did have detectable chrysoeriol, a precursor of tricin, in the pericarp samples. We also used RNA-Seq analysis of developing pericarp and embryo samples of the two cultivars to compare the expression of genes involved in the flavonoid biosynthetic pathway. The results presented here suggest that understanding the basis of tricin accumulation in rice pericarp may lead to an approach to increasing tricin levels in whole grain rice. From analysis of gene expression levels in the pericarp samples it appears that regulation of the flavone specific genes is independent of regulation of the anthocyanin biosynthetic genes. It therefore may be feasible to develop brown pericarp rice cultivars that accumulate tricin in the pericarp.

## Introduction

Rice (*Oryza sativa*) is the world’s most important food crop and is the primary food for more than half of the world’s population (Gnanamanickam, 2009). However, most rice is consumed as white rice in which the bran (pericarp, testa, aleurone, and embryo) has been removed. The remaining endosperm is mainly composed of starch. Nutrients found in the bran include vitamins, fiber and the antioxidant γ-oryzanols, phenolics, and flavonoids (Min, McClung & Chen, 2011; Esa, Ling & Peng, 2013; Burlando & Cornara, 2014). A metabolomics analysis of rice bran identified 453 phytochemicals, 65 of which have been associated with health benefits (Zarei et al., 2017). Vitamin B1 deficiency has been associated with diets in which white rice is a major component (Whitfield et al., 2017). Currently there are efforts to incorporate nutrient rich rice bran into human diets, particularly in developing nations (Friedman, 2013; Borresen & Ryan, 2014). The bran by-product of rice milling has been used in livestock feed for over 100 years and is also used in pet foods (Ryan, 2011).

Rice bran also contains flavonoids that have been found to have disease preventative effects. The methylated flavone tricin has been reported to have anticancer activity. Dietary tricin resulted in reductions in intestinal and colon cancers in mice (Cai et al., 2005; Oyama et al., 2009). Tricin acts by suppressing inflammatory responses and inhibiting COX-2 enzymes (Desai, Prickril & Rasooly, 2018). Methylated flavones have been reported to have higher bioavailability than similar unmethylated compounds (Walle, 2007; Walle et al., 2007). The degree of flavone *O*-methylation was correlated with gastrointestinal cancer chemopreventive efficacy in mice, where pentamethyoxyflavone > tricin > apigenin (Cai et al., 2009).

Tricin has been reported from a wide range of plant species and is found in many different tissues (Wollenweber & Dorr, 2008; Zhou & Ibrahim, 2010; Li et al., 2016). However, whole cereal grains, such as rice, barley, oat, and wheat, are the only food sources of tricin, which is located in the bran portion of the grain (Goufo et al., 2015; Nakano et al., 2011; Zhang et al., 2012; Moheb et al., 2013).

Rice cultivars vary in the color of the pericarp in the unpolished grain, which can be light brown, brown, red, or purple (commonly called black). The colors of red and purple pericarp rice grains are due to the presence of proanthocyanidins and anthocyanins, respectively. Most of the rice grown for consumption is of the light brown and brown pericarp types, and due to cultural preferences, most is milled to produce white rice (Mohan et al., 2017). Red and purple rices are sold as specialty rices that may have health benefits attributable to their high antioxidant levels (Min, McClung & Chen, 2011). Variation in tricin levels was found in bran from rice genotypes with light brown, brown, red, and purple pericarp color, with the purple pericarp genotypes having the highest levels of tricin (Poulev et al., 2018).

**Figure 1:**
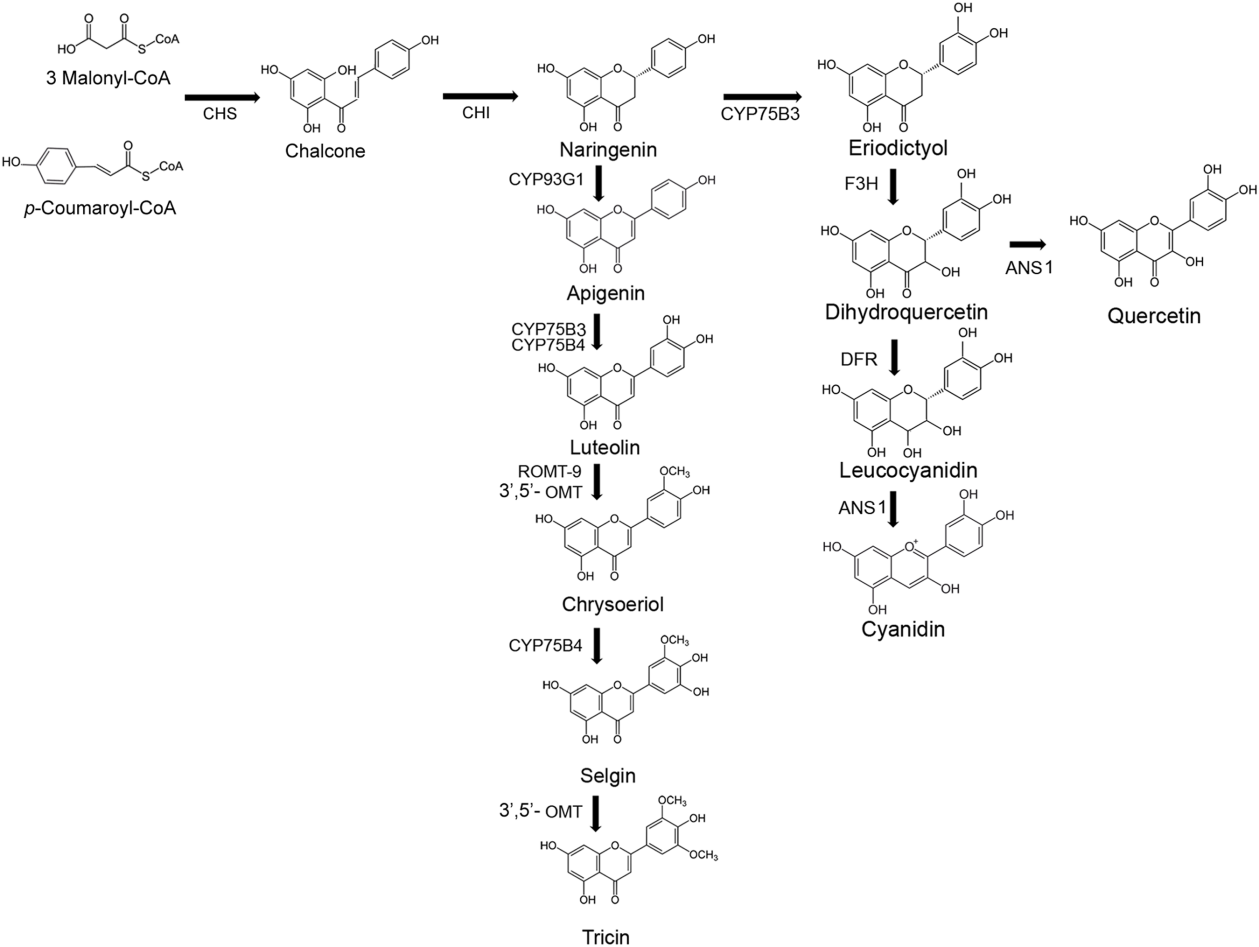
Flavone and anthocyanin biosynthetic pathways. Specific rice gene identifications, where known, are presented in Table 3. ANSI, anthocyanin synthase I; CHS, chalcone synthase; CHI, chalcone isomerase; DRF, Dihydroflavonol-4-reductase; F3H, Flavanone 3-hydroxylase; OMT, *O*-methyltransferase.

Much is known about the biosynthesis of flavones in plants (Jiang, Doseff & Grotewold, 2016) and most steps in the pathway to tricin have been established (Lam, Liu & Lo, 2015). In both rice and sorghum the lignin biosynthetic enzyme COMT (5-hydroxyconiferaldehyde *O*-methyltransferase = caffeate *O*-methyltransferase) has been shown to be involved in tricin biosynthesis but another, as yet unidentified, *O*-methyltransferase is likely also involved (Lam, Liu & Lo, 2015; Eudes et al., 2017). Understanding the tricin biosynthetic pathway and its regulation is of increasing interest since the discovery that tricin is a component of lignin, mainly in monocots (Lan et al., 2016). The proposed tricin biosynthetic pathway is shown in Fig. 1.

Here, we used metabolite analysis and RNA-Seq analysis to determine the basis for the higher levels of tricin in a purple pericarp rice genotype. We analyzed tricin levels in developing pericarp and embryo samples of a purple pericarp genotype, IAC600, that had high tricin levels in the bran and a light brown pericarp genotype, Cocodrie, that had no detectable tricin in the bran (Poulev et al., 2018). The high tricin content in the bran of IAC600 could be attributed to the synthesis of tricin in both the pericarp and embryo, whereas in Cocodrie, tricin was detected only in the embryo samples, which can explain the lack of detectable tricin in the bran as reported previously. We also used RNA-Seq analysis of developing pericarp and embryo samples of the two cultivars to compare the expression of genes involved in the flavonoid biosynthetic pathway.

## Materials and Methods

### Plant materials

Seeds of the rice cultivars Cocodrie and IAC600 were provided by the USDA Dale Bumpers National Rice Research Center, Stuttgart, AR. Cocodrie is a tropical japonica light brown pericarp cultivar developed by the Louisiana State University (Linscombe et al., 2000). IAC600 is a temperate japonica purple pericarp cultivar that was developed in Brazil (Forster et al., 2013). Seeds were imbibed overnight in water and then planted in 2.5 × 3.8 cm cells filled with Turface MVP (Profile Products LLC, Buffalo Grove, IL, USA). The inserts were held in a tray filled with water that came to just above the Turface level. When the seedlings were 8–10 cm tall they were transferred to 10 × 10 cm pots containing Turface for growth in the greenhouse or transplanted to the field.

For growth in the greenhouse, the pots were placed in 60 cm long × 30 cm wide × 33 cm high plastic tubs filled with a fertilizer solution that came to just above the Turface level in the pots. The fertilizer solution was composed of five mL of Peters 15-15-15 water soluble fertilizer (ICL Specialty Fertilizers, Dublin, OH, USA), 2.5 mL of Sprint 330 (Iron Chelate) (BASF, Florham Park, NJ, USA), and 30 mL of ZeroTol 2.0 algaecide/fungicide (BioSafe Systems, LLC, East Hartford, CT, USA) in 15 L of water. The fertilizer solution was aerated with aquarium pumps and was replaced weekly.

Plants were also grown in the field at the Rutgers University Horticulture Farm III, East Brunswick, New Jersey. Plants of each cultivar were grown in a 1.8 m long × 0.9 m wide × 0.3 m deep raised beds lined with a heavy plastic pond liner. The raised beds were filled with a mixture of field soil (Sassafras sandy loam) and compost, supplemented with 150 mL Wollastonite (calcium silicate). Wollastonite was added as a soil amendment since rice has a high demand for silicon (Tubaña & Heckman, 2015). The water level in the raised beds was maintained slightly above the soil surface. Miracle-Gro All Purpose Plant Food (Scotts Miracle-Gro, Marysville, OH, USA) was applied every 2–3 weeks in accordance with directions on the product label.

### Tissue extraction

For metabolite analysis embryo and pericarp samples from greenhouse and field grown plants were harvested at 7, 10, and 14 days after anthesis. Extraction of free polyphenols from the embryo and pericarp samples was modified from that reported by Goufo et al. (2014). Triplicate extracts for each sample were prepared with each replicate composed of samples from multiple plants. The hulls were removed from the developing grains and the embryos and pericarps were separated. Each replicate consisted of embryos or pericarps from 20 developing rice grains. The embryo samples were ground in a microfuge tube in 400 μL of 70% methanol. The pericarp samples were ground to a powder in a mortar with liquid nitrogen. The powder was transferred to a microfuge tube and one mL of 70% methanol was added. The embryo and pericarp samples in 70% methanol were incubated at 70 °C for 30 min. The samples were centrifuged for 5 min at maximum speed in a microfuge and the supernatants transferred to a new tube. The supernatants were placed at −20 °C overnight. The following day the samples were centrifuged again to remove any remaining particulates. Ten μL samples were injected into the UPLC/mass spectrometer (MS) system.

### LC/MS analysis of rice embryo and pericarp tissues

Samples were separated and analyzed by a UPLC/MS system including the Dionex® UltiMate 3000 RSLC ultra-high pressure liquid chromatography system, consisting of a workstation with ThermoFisher Scientific’s Xcalibur v. 4.0 software package combined with Dionex^®^’s SII LC control software, solvent rack/degasser SRD-3400, pulseless chromatography pump HPG-3400RS, autosampler WPS-3000RS, column compartment TCC-3000RS, and photodiode array detector DAD-3000RS. After the photodiode array detector the eluent flow was guided to a Q Exactive Plus Orbitrap high-resolution high-mass-accuracy MS. Mass detection was full MS scan from 100 to 1,000 m/z in either positive, or negative ionization mode with electrospray (ESI) interface. Sheath gas flow rate was 30 arbitrary units, auxiliary gas flow rate was 7, and sweep gas flow rate was 1. The spray voltage was 3,500 V (−3,500 for negative ESI) with a capillary temperature of 275 °C. The mass resolution was 140,000. Substances were separated on a Phenomenex^TM^ Kinetex C8 reverse phase column, size 100 × 2 mm, particle size 2.6 mm, pore size 100 Å. The mobile phase consisted of two components: Solvent A (0.5% ACS grade acetic acid in LCMS grade water, pH 3–3.5), and Solvent B (100% acetonitrile, LCMS grade). The mobile phase flow was 0.20 mL min^−1^, and a gradient mode was used for all analyses. The initial conditions of the gradient were 95% A and 5% B; by 30 min the proportion reached 5% A and 95% B which was kept for the next 8 min, and during the following 4 min the ratio was brought to initial conditions. An 8 min equilibration interval was included between subsequent injections. The average pump pressure using these parameters was typically around 3,900 psi for the initial conditions.

Putative formulas were determined by performing isotope abundance analysis on the high-resolution mass spectral data with Xcalibur v. 4.0 software and reporting the best fitting empirical formula. Database searches were performed using reaxys.com (RELX Intellectual Properties SA, Amsterdam, The Netherlands) and SciFinder (American Chemical Society, Washington, DC, USA). The databases were reviewed for compounds identified from the analyzed rice samples with molecular masses corresponding to the LC-FTMS data. Any matches were investigated by comparing the literature and the experimental data; putative compound assignments were made when matches were identified.

Tricin (m/z 331.07–331.09, +ESI) was identified and quantitated by comparison to a tricin standard from Dalton Research Molecules, Toronto, Canada. Free tricin was quantitated based on a UV spectrum standard curve measured at 351 nm. Tricin-*O*-hexoside (m/z 493, +ESI), tricin-*O*-deoxyhexoside-*O*-hexoside (m/z 639.19–639.20, +ESI), chrysoeriol (m/z 301.07–301.08), and chrysoeriol-7-*O*-rutinoside (m/z 609.1–609.2) were identified based on published mass spectral data (Galland et al., 2014) and quantitated as tricin equivalents based on the tricin standard curve. Statistical analysis of the data was done by using the Prism 4 program (GraphPad Software, San Diego, CA, USA).

### RNA isolation

For RNAseq analysis, RNA was extracted from embryo and pericarp samples from field grown plants at 10 days after anthesis. RNA was isolated by using the Synergy 2.0 Plant DNA isolation kit (Ops Diagnostics, Lebanon, NJ, USA) for tissue homogenization and the Zymo Fungal/Bacterial RNA MiniPrep kit (Zymo Research Corp, Irvine, CA, USA) for RNA purification. RNA was isolated from 6 to 20 embryos and two pericarps per extraction. Higher amounts of pericarp tissue were not completely homogenized in the bead beater. Tissue samples were dissected from the developing rice grains and were dropped into the Synergy pre-filled homogenization tubes containing a grinding matrix, and maintained in dry ice until extraction. A total of 800 μL of the Plant Homogenization Buffer, a CTAB-based buffer, supplemented with 16 μL of β-mercaptoethanol, was added to each sample tube. The samples were homogenized by using a HT Mini bead beater (Ops Diagnostics, Lebanon, NJ, USA) for 3 min. The homogenized samples were centrifuged to remove debris, 600 μL of the supernatant was transferred to a Zymo-Spin IIIC column, and the RNA miniprep kit protocol followed for the rest of the RNA isolation. RNA from multiple extractions was pooled for library preparation.

### Library preparation and sequencing

RNA-Seq was performed at the Waksman Genomics Core Facility, Rutgers University. In brief, RNA concentration and integrity was verified using BioAnalyzer 2100 with RNA 6000 Nano Labchips according to manufacturer’s instructions (Agilent Technologies, Santa Clara, CA, USA). RNA samples had 28 S/18 S ratios ranging from 1.8 to 2.0 and RIN (RNA Integrity Number) values of between 7.0 and 9.0. PolyA RNA was isolated from 5 to 10 μg total RNA with oligo(dT) beads using two rounds of oligo-dT purification and 50–100 ng mRNA was used for Illumina library preparation. The libraries were prepared from each of three biological RNA replicates from 10-day embryo and pericarp samples from field grown Cocodrie and IAC600 plants.

The directional cDNA libraries were prepared using the dUTP method with a NEB Ultra Directional RNA Library Prep kit for Illumina (New England Biolabs Inc., Ipswich, MA, USA). Each sample was ligated with different indexes and amplified with 12 PCR cycles. The quality and quantity of cDNA libraries were evaluated using Qubit 2.0 (Invitrogen; Life Technologies, Carlsbad, CA, USA), BioAnalyzer 2100 with DNA 1000 kit (Agilent Technologies, Santa Clara, CA, USA) and Real-time PCR using KAPA Library Quantification Kit (Kapa Biosystems, Wilmington, MA, USA). Adapter-ligated cDNA fragment libraries were pooled together and loaded into an Illumina NextSeq500 using NextSeq® 500/550 High Output Kit version 3 according to the manufacturer’s protocol (Illumina, San Diego, CA, USA).

Raw data samples were de-multiplexed and FastQC software (v0.11.4) was applied for the quality control of raw sequences. Fastq output files were used for all downstream applications. Adaptor sequences and reads shorter than 36 bp and/or reads with a quality score lower than 15 were removed from the dataset using Cutadapt software (v1.3).

### Accession number

The sequence data is available at NCBI under the BioProject ID PRJNA490738.

### Sequence analysis

The processed RNA-Seq reads were imported into CLC Genomics Workbench (Version 10.0, Qiagen Bioinformatics, Germantown, MD, USA) and trimmed to remove low-quality reads with the following parameters: minimum quality score 0.05 and maximum number of ambiguities 2. After trimming the average read length of all samples was greater than 74 bases. The trimmed reads were mapped to the *Oryza sativa* Nipponbare reference genome IRGSP-1.0 (Kawahara et al., 2013) transcripts using the RNA-Seq analysis tool, with the mapping settings set to minimum length fraction (0.8) and minimum similarity fraction (0.8). Comparison of gene expression between pairs of samples was done using the CLC Differential Expression for RNA-Seq tool.

## Results

### Levels of tricin and tricin derivatives in developing embryo and pericarp

**Figure 2:**
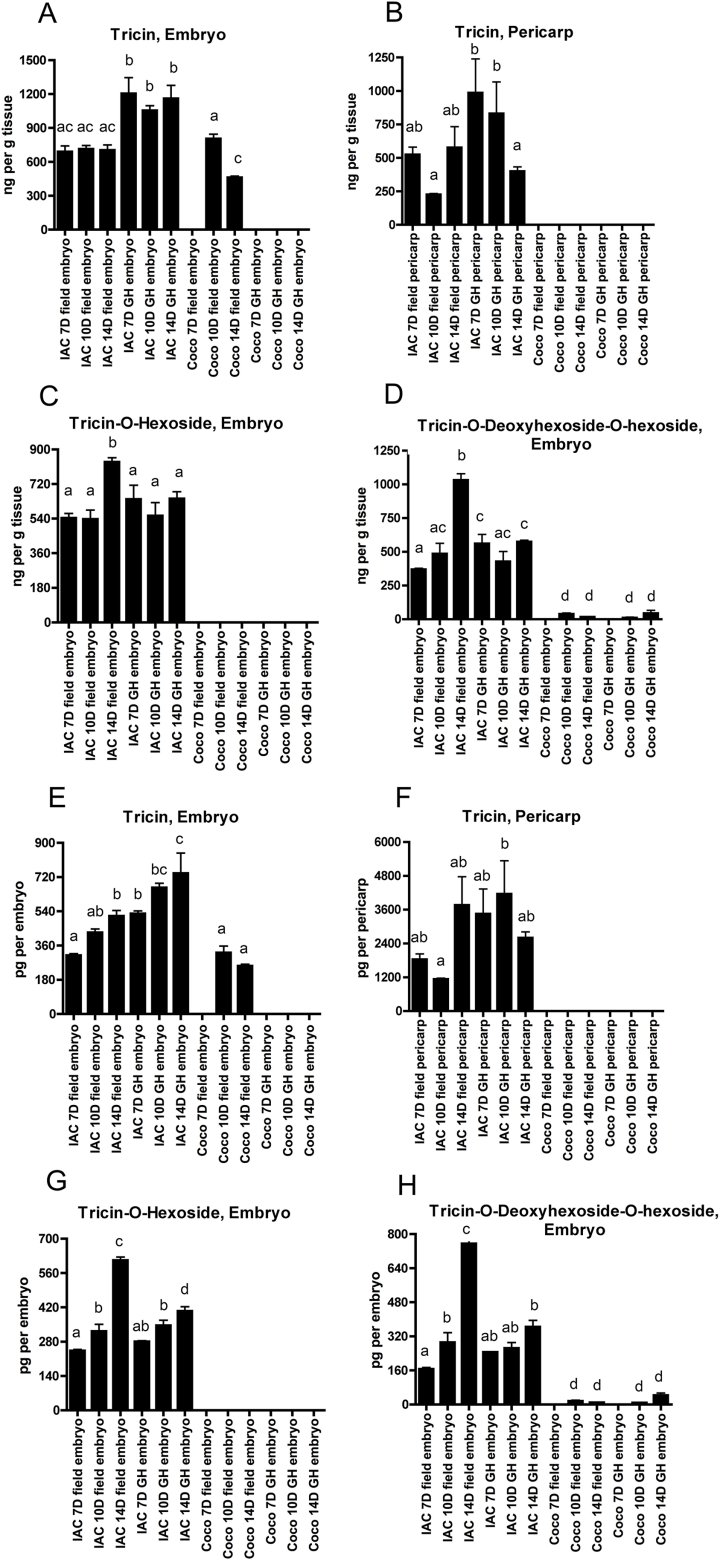
Tricin and tricin derivative levels in embryo and pericarp tissue of developing grains of rice cultivars IAC600 (IAC) and Cocodrie (Coco) grown in the field (F) or in the greenhouse (GH). (A–D) Tricin and tricin derivative levels expressed as ng per gram of tissue. (E–H) Tricin and tricin derivative levels expressed as pg per embryo or pg per pericarp sample. The data presented are the means and standard deviations of three biological replicates. Columns with different letters indicate a significant difference in compound levels (*p* < 0.05, one way ANOVA with Tukey’s Multiple Comparison Test). To better visualize the variation among the samples in amounts of specific compounds, the *y*-axes of the individual graphs are in different scales.

Free tricin was detected in all the field and greenhouse grown IAC600 embryo and pericarp samples (Fig. 2). It was also detected in the 10 and 14-day field grown Cocodrie embryo samples but was not detected in the Cocodrie green house grown embryo samples or any of the Cocodrie pericarp samples. The tricin derivatives, tricin-*O*-hexoside and tricin-*O*-deoxyhexoside-hexoside, were also detected in all the IAC600 embryo samples and tricin-*O*-deoxyhexoside-hexoside was detected in the 10 and 14 day field grown and green house grown Cocodrie embryo samples. The tricin derivatives were not detected in any of the IAC600 or Cocodrie pericarp samples. Apparently the IAC600 pericarp tissues, which can synthesize free tricin, are not capable of converting it to the glycoside derivatives, which can be detected in both the IAC600 and Cocodrie embryo samples. Lignin-bound tricin would not be detected in our analysis. The flavonoids quercetin-3-*O*-glucoside and cyanidin-3-*O*-glucoside were detected exclusively in the IAC600 pericarp samples. Table 1 summarizes the detection of the compounds in the developing embryo and pericarp samples of the two cultivars, which vary in pericarp color.

**Table 1:** Summary of compounds detected (yes) or not detected (no) in developing rice embryo (E) and pericarp tissues (P).

	IAC600-E	IAC600-P	Cocodrie-E	Cocodrie-P
Tricin	Yes	Yes	Yes	No
Tricin derivatives	Yes	No	Yes	No
Chrysoeriol	Yes	Yes	Yes	Yes
Chrysoeriol-7-*O*-rutinoside	Yes	No	Yes	No
Quercetin-3-glucoside	No	Yes	No	No
Cyanidin-3-*O*-glucoside	No	Yes	No	No

The cultivars IAC600 and Cocodrie were chosen for comparison because in a previous study comparing bran tricin and tricin-*O*-hexoside levels among 20 rice cultivars varying in pericarp color, both were found at a relatively high level in IAC600 but were not detected in Cocodrie (Poulev et al., 2018). The results reported here are consistent with the previous study. Free tricin detected in the bran of IAC600 would be originating from both the embryo and the pericarp, whereas the tricin derivatives would be originating from the embryo since here they were not detected in the pericarp sample. The total levels of free tricin plus tricin derivatives in the IAC600 embryo samples were generally higher than the level of free tricin in the pericarp samples when expressed as ng per gram fresh weight of tissue (Fig. 2A). However, the IAC600 pericarp fraction overall has considerably more free tricin than the tricin plus tricin derivatives in the embryo fraction because it overall has more mass than the embryo fraction. When expressed as amount per embryo or per pericarp, there was more free tricin in the pericarp samples than in the embryo fractions (Fig. 2B). Also, the level of the free tricin in the IAC600 pericarp was higher than the combination of free tricin plus tricin derivatives in the embryo fraction. Overall, on a per grain basis, most of the total tricin component in IAC600 would be originating from the free tricin in the pericarp.

Here tricin was detected only in the embryo samples of the cultivar Cocodrie and not in the pericarp samples. The previous lack of detection of tricin or tricin-*O*-hexoside in the bran of Cocodrie (Poulev et al., 2018) is likely due to the overall small contribution of the embryo fraction to the bran. The results obtained here with the Cocodrie embryo and pericarp samples are similar to those reported for another light brown pericarp cultivar, Nipponbare, where tricin and tricin derivatives were detected only in the embryo samples and not in the pericarp (Galland et al., 2014).

There were differences in tricin and tricin derivative levels between the field grown and greenhouse grown samples. For the IAC600 samples, there were generally more or similar levels detected in the greenhouse samples relative to the field grown samples. However, for the Cocodrie embryo samples, free tricin was only detected in the field grown samples and not in the greenhouse grown samples but tricin-*O*-deoxyhexoside-*O*-hexoside was detected in both the field and greenhouse grown samples. It is generally considered that the variations in flavonoid composition among rice cultivars are mainly genetic (Chen, McClung & Bergman, 2016; Matsuda et al., 2012), as seen here in the contrast in flavonoid composition between the IAC600 and Cocodrie pericarp samples. There may also be an environmental component that influences the levels of particular flavonoids, as seen here in the variations within the two cultivars between field grown and greenhouse grown samples.

**Figure 3:**
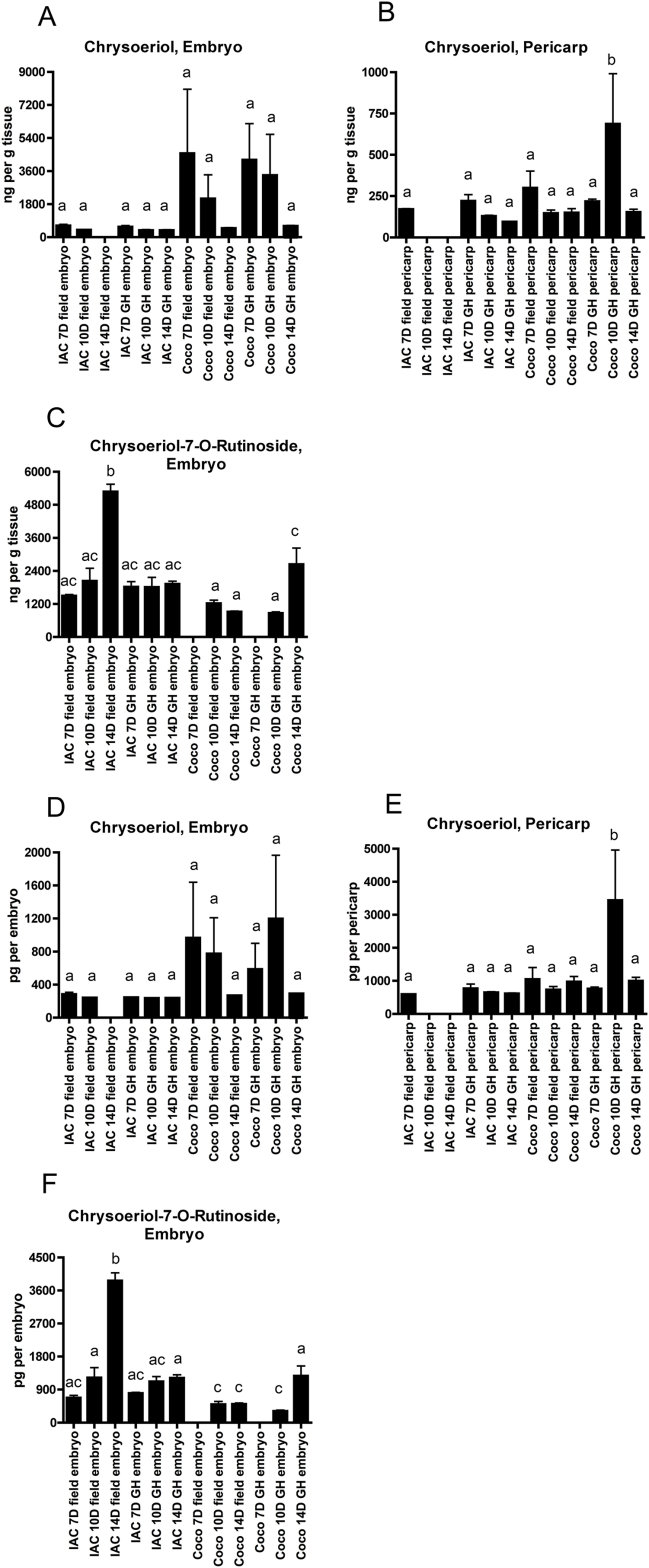
Chrysoeriol and chrysoeriol-7-*O*-rutinoside levels in embryo and pericarp tissue of developing grains of rice cultivars IAC600 (IAC) and Cocodrie (Coco) grown in the field (F) or in the greenhouse (GH). (A–C) Chrysoeriol and chrysoeriol-7-*O*-rutinoside levels expressed as ng per gram of tissue. (D–F) Chrysoeriol and chrysoeriol-7-*O*-rutinoside levels expressed as pg per embryo or pg per pericarp sample. The data presented are the means and standard deviations of three biological replicates. Columns with different letters indicate a significant difference in compound levels (*p* < 0.05, one way ANOVA with Tukey’s Multiple Comparison Test). To better visualize the variation among the samples in amounts of specific compounds, the *y*-axes of the individual graphs are in different scales.

Although no free tricin or tricin derivatives were detected in the Cocodrie pericarp samples, a precursor of tricin, chrysoeriol, was detected in all the Cocodrie pericarp samples as well as most of the IAC600 pericarp samples (Fig. 3). These results differ from the previous report on the cultivar Nipponbare where chrysoeriol was detected only in the embryo fraction and not in the pericarp fraction (Galland et al., 2014). Chrysoeriol and the glycoside derivative chrysoeriol-7-*O*-rutinoside were also detected in most of the Cocodrie and IAC600 embryo samples. Chrysoeriol-7-*O*-rutinoside was not detected in any of the pericarp samples. In the tricin biosynthetic pathway chrysoeriol is converted to selgin, the immediate precursor to tricin (Fig. 1). Selgin was not detected in our samples. Overall these results suggest that the lack of detectable free tricin in the Cocodrie pericarp samples is due to a lack of conversion of chrysoeriol to free tricin. It may be that any tricin produced in the Cocodrie pericarp was bound to lignin, which would not be detected here. Also, although free tricin and/or chrysoeriol were detected in the pericarp samples of both cultivars, no glycoside derivatives of either flavone were detected in the pericarp but were detected in the embryo samples. Apparently the pericarp tissues are not able to synthesize the glycoside derivatives of tricin or chrysoeriol.

### Differential gene expression of flavone biosynthetic genes between Cocodrie and IAC600 developing embryo and pericarp samples

RNA was isolated from 10 day developing embryo and pericarp field grown Cocodrie and IAC600 plants and was subjected to RNA-Seq (Mortazavi et al., 2008). The characteristics of the sequences obtained are summarized in Table 2. We obtained between 61 and 96 million useable reads per sample.

**Table 2:** Overview of mapped RNA-seq reads.

Sample	Trimmed reads, million	Reads mapped, million (%)
Cocodrie embryo 1	64.4	56.0 (86.9)
Cocodrie embryo 2	78.4	66.8 (85.2)
Cocodrie embryo 3	79.6	70.9 (89.0)
Cocodrie pericarp 1	80.7	75.4 (93.4)
Cocodrie pericarp 2	96.6	89.9 (93.0)
Cocodrie pericarp 3	61.2	56.7 (92.6)
IAC600 embryo 1	83.6	57.9 (69.2)
IAC600 embryo 2	73.7	67.5 (91.5)
IAC600 embryo 3	80.0	76.5 (95.6)
IAC600 pericarp 1	77.1	72.9 (94.5)
IAC600 pericarp 2	79.5	75.2 (94.5)
IAC600 pericarp 3	66.2	62.5 (94.9)

The reads were mapped to the annotated transcripts of the rice cultivar Nipponbare and were analyzed pairwise for differential gene expression between IAC600 pericarp and IAC600 embryo, IAC600 pericarp and Cocodrie pericarp, IAC600 embryo and Cocodrie embryo, and Cocodrie pericarp and Cocodrie embryo. The data was analyzed for expression of genes involved in flavonoid biosynthesis. Flavonoid biosynthesis has been intensively studied for many years, and the basic pathway has been established (Tohge, Perez De Souza & Fernie, 2017). The specific rice genes involved in many of the biosynthetic steps are known and are summarized in Table 3 with their gene numbers assigned by the Rice Annotation Project Database and by the MSU Rice Genome Annotation Project, since previous publications vary as to the annotation system used. Here, we use the gene designations assigned by the Rice Annotation Project Database. Of particular interest in this study are the comparisons of the IAC600 and Cocodrie pericarp samples, since there is a plus/minus distinction between those samples regarding the presence of detectable tricin. Table 4 summarizes the differential gene expression data for the genes known to be involved in flavonoid biosynthesis.

**Table 3:** Rice flavonoid pathway genes.

Enzyme activity	Gene name	
	Rice annotation project (RAP)*	MSU rice genome annotation project**
Early pathway genes		
Chalcone synthase	Os11g0530600	LOC_Os11g32650
Chalcone isomerase	Os03g0819600	LOC_Os03g60509
Tricin biosynthesis		
Flavone synthase II (CYP93G1)	Os04g0101400	LOC_Os04g01140
Flavonoid 3′-hydroxylase (CYP75B3)	Os10g0320100	LOC_Os10g17260
Flavone 3′,5′-hydroxylase (CYP75B4)	Os10g0317900	LOC_Os10g16974
3′-*O*-methyltransferease (ROMT9/COMT)	Os08g0157500	LOC_Os08g06100
3′,5′-*O*-methyltransferase*** (ROMT15)	Os08g0498100	LOC_Os08g38900
3′,5′-*O*-methyltransferase*** (ROMT17)	Os08g0498400	LOC_Os08g38910
Anthocyanin biosynthesis		
Flavanone 3-hydroxylase	Os04g0662600	LOC_Os04g56700
Flavonoid 3′-hydroxylase (CYP75B3)	Os10g0320100	LOC_Os10g17260
Dihydroflavonol reductase	Os01g0633500	LOC_Os01g44260
Anthocyanin synthase I	Os01g0372500	LOC_Os01g27490
Regulatory gene		
*Kala4*	Os04g0557500	LOC_Os04g47059

**Notes:**

* http://rapdb.dna.affrc.go.jp/tools/converter/run.

** http://rice.plantbiology.msu.edu/index.shtml.

*** Not confirmed as involved in tricin biosynthesis in rice.

**Table 4:** Differential gene expression between IAC600 and Cocodrie pericarp (P) and embryo (E) samples.

	IAC600-P vs IAC600-E	IAC600-P vs Coco-P	IAC600-E vs Coco-E	Coco-P vs Coco-E
Early pathway genes				
Chalcone synthase				
Maximum group mean	10,445	10,445	19	16
Fold change	478	716	1.9	1.2
Log2 fold change	8.9	9.5	0.92	0.31
*p*-value	0	0	0	0.009
Chalcone isomerase				
Maximum group mean	400	400	138	138
Fold change	2.8	7.8	1	−2.8
Log2 fold change	1.5	3	0.01	−1.5
*p*-value	0	0	0.88	0
Tricin pathway genes				
Flavone synthase II (CYP93G1)				
Maximum group mean	1.1	0.9	1.1	0.3
Fold change	−1.4	18.2	3.6	−7.4
Log2 fold change	−0.4	4.2	1.9	−2.9
*p*-value	0.18	6.10*E*-13	1.50*E*-08	4.80*E*-07
Flavonoid 3′-hydroxylase (CYP75B3)				
Maximum group mean	1,466	1,466	1.7	4.7
Fold change	733	346	2.4	5
Log2 fold change	9.5	8.4	1.2	2.3
*p*-value	0	0	1.00*E*-06	0
Flavonoid 3′,5′-hydroxylase (CYP75B4)				
Maximum group mean	3.8	11.4	2.5	11.4
Fold change	2.3	−2.6	−1.4	4.1
Log2 fold change	1.2	−1.4	−0.5	2
*p*-value	1.30*E*-05	1.20*E*-12	0.05	0
3′-*O*-methyltransferase (ROMT9/COMT)				
Maximum group mean	227	280	5.3	280
Fold change	35.9	−1.1	1.3	49.6
Log2 fold change	5.2	−0.2	0.3	5.6
*p*-value	0	0.25	0.02	0
3′,5′-*O*-methyltansferase (ROMT15)				
Maximum group mean	207	207	16	170
Fold change	11.1	1.3	1.4	11.1
Log2 fold change	3.5	0.4	0.5	3.5
*p*-value	0	3.30*E*-03	5.00*E*-06	0
3′,5′-*O*-methyltansferase (ROMT17)				
Maximum group mean	27	66	27	66
Fold change	−4.3	−8.3	3.6	6.9
Log2 fold change	−2.1	−3.05	1.87	2.8
*p*-value	0	0	0	0
Cyanidin pathway genes				
Flavanone 3-hydroxylase (F3H)				
Maximum group mean	2,584	2,584	3.8	6.7
Fold change	586	431	3.3	4.5
Log2 fold change	9.2	8.7	1.7	2.2
*p*-value	0	0	6.70*E*-12	0
Dihydroflavonol-4-reductase (DFR)				
Maximum group mean	4,201	4,201	4.1	6.7
Fold change	912	698	3.6	4.8
Log2 fold change	9.8	9.4	1.8	2.3
*p*-value	0	0	2.20*E*-16	0
Anthocyanin synthase, (ANS1)				
Maximum group mean	5,876	5,876	3.6	6.2
Fold change	1,416	1,047	3	3.9
Log2 fold change	10.5	10	1.6	2
*p*-value	0	0	1.80*E*-14	0
Regulatory gene				
*Kala4*				
Maximum group mean	225	225	0.3	0.4
Fold change	754	629	7.1	7.4
Log2 fold change	9.6	9.3	2.8	2.9
*p*-value	0	0	4.00*E*-06	8.90*E*-07

**Note:**

The maximum group mean is the mean RPKM (reads per kilobase of exon model per million mapped reads) of the samples with the highest mean RPKM in the pairwise comparison. Fold change is the ratio of the mean expression values of the samples in the comparison. Positive fold change values indicate the maximum group mean value is the mean from the first tissue in the pairwise comparison and negative fold change values indicate it is from the second tissue in the pairwise comparison. The statistical model used by the analysis program corrects for differences in library size so the fold changes cannot be determined by simple algebraic calculations of the maximum group means.

The first committed step in flavonoid biosynthesis is catalyzed by the enzyme chalcone synthase in which *p*-coumaroyl-CoA and three molecules of malonyl-CoA are condensed to form chalcone. In rice, chalcone synthase is a member of a gene family composed of 17–26 genes (Han et al., 2009). Sequence duplication and divergence has resulted in functional divergence of the encoded proteins, with genes on chromosomes 7 and 11 being considered the ones likely involved in flavonoid biosynthesis (Han et al., 2009). Functional complementation of the Arabidopsis chalcone synthase *tt4* mutant confirmed the activity of the rice chalcone synthase gene on chromosome 11 (Os11g0530600) (Shih et al., 2008). Chalcone synthase was highly up-regulated in the IAC600 pericarp relative to the IAC600 embryo and Cocodrie pericarp expression levels, at 8.9 and 9.5 log2-fold changes, respectively (Table 4). In fact, chalcone synthase was the second most highly expressed transcript in the IAC pericarp samples. Chalcone synthase in the IAC600 embryo samples was slightly up-regulated relative to the Cocodrie embryo samples, and it was up-regulated in the Cocodrie pericarp samples relative to the Cocodrie embryo samples. The levels of expression of chalcone synthase in the IAC600 embryo and the Cocodrie embryo and pericarp samples were all considerably lower than in the IAC600 pericarp samples.

Chalcone isomerase (Os03g0819600) catalyzes the conversion of chalcone to naringenin. Functional complementation of the Arabidopsis chalcone isomerase *tt5* mutant confirmed the activity of the rice chalcone isomerase gene (Shih et al., 2008). Chalcone isomerase was up-regulated in the IAC600 pericarp relative to the IAC600 embryo and Cocodrie pericarp expression levels, at 1.5 and 3 log2-fold changes, respectively. There was no difference in expression between the two embryo samples and the Cocodrie embryo sample had a higher level of expression than the Cocodrie pericarp sample.

The desaturation of the flavanone naringenin to the flavone apigenin is the first committed enzymatic step in the flavone biosynthetic pathway, catalyzed by flavone synthase II. Flavone synthase activity of a cytochrome P450 monooxygenase, CYP93G1 (FNSII, Os04g0101400), was confirmed in rice seedlings by in vitro enzyme assays and absence of tricin detection in a T-DNA insertion mutant (Lam et al., 2014). When CYP93G1 was expressed in yeast it was able to desaturate both naringenin and eriodictyol to apigenin and luteolin, respectively (Lam et al., 2014). However, in a T-DNA mutant of CYP93G1, naringenin but not eriodictyol accumulated, indicating that naringenin was the likely substrate in planta (Lam et al., 2014). There was no significant difference in the level of CYP93G1 between the IAC600 pericarp and embryo samples. CYP93G1 levels were up-regulated in the IAC600 pericarp relative to the Cocodrie pericarp sample. CYP93G1 was more highly expressed in the IAC600 embryo relative to the Cocodrie embryo and in the Cocodrie embryo relative to the Cocodrie pericarp.

The conversion of apigenin to tricin requires two hydroxylations and two methylations. Two cytochrome P450 enzymes are considered to catalyze the hydroxylation steps. CYP75B3 (Os10g0320100) was confirmed as a flavonoid 3′-hydroxylase by expression in an Arabidopsis *tt7* mutant in which the flavonol quercetin was accumulated instead of kaempferol (Shih et al., 2008). When expressed in yeast microsomes CYP75B3 had similar activity with both the flavanone naringenin and the flavone apigenin (Park et al., 2016). CYP75B4 (Os10g0317900) was shown to have both flavonoid 3′ and 5′-hydroxylase activity, acting on both apigenin and chrysoeriol (Lam, Liu & Lo, 2015). In a CYP75B4 knockout line, higher levels of luteolin, but no tricin were detected, indicating that CYP75B3 likely did function in the flavone pathway as well as the anthocyanin pathway for 3′ hydroxylation, but CYP75B4 was required for the 5′ hydroxylation in the production of tricin (Lam, Liu & Lo, 2015).

CYP75B3 was highly overexpressed in the IAC600 pericarp relative to the IAC600 embryo and the Cocodrie pericarp samples, at 9.5 and 8.4 log2 fold-change, respectively. This is consistent with its proposed role in 3′-hydroxylation of naringenin in the pathway for cyanidin synthesis, which is unique to the IAC600 pericarp tissue. CYP75B3 was moderately up-regulated in the IAC600 embryo relative to the Cocodrie embryo samples and in the Cocodrie pericarp relative to the Cocodrie embryo samples.

CYP75B4 was moderately over expressed in the IAC600 pericarp relative to the IAC600 embryo samples, but the overall expression level was considerably lower than that of CYP75B3. It was more highly expressed in the Cocodrie pericarp than in the IAC600 pericarp samples and in the Cocodrie embryo than in the IAC600 embryo samples. It was more highly expressed in the Cocodrie pericarp than in the Cocodrie embryo samples.

The 3′-*O*-methylation of luteolin to form chrysoeriol and the 5′-*O*-methylation of selgin to form tricin are likely carried out by more than one *O*-methyltransferase. In a T-DNA insertion line that disrupted the expression of an *O*-methyltransferase designated ROMT-9, tricin levels in seedlings were reduced to 46% of that of wild-type seedlings (Lam, Liu & Lo, 2015). The results from the T-DNA insertion line indicate ROMT-9 is involved in tricin biosynthesis, likely along with one or more additional as yet unidentified OMTs. ROMT-9 was reported to methylate the 3′OH of flavonoid substrates including luteolin, the substrate for the first methylation in tricin biosynthesis (Kim et al., 2006). ROMT9 is the same rice gene (Os08g0157500) that was shown to also function as 5-hydroxyconiferaldehyde *O*-methyltransferase, designated OsCOMT1, in the syringyl lignin biosynthetic pathway (Koshiba et al., 2013). In a rice plant in which expression of OsCOMT1 was down-regulated through RNAi, the syringyl lignin content was reduced. The rice gene ROMT9/OsCOMT1 thus functions both in the lignin and tricin biosynthetic pathways. The similar sorghum gene, SbCOMT, was also shown to function both in the syringyl lignin and tricin biosynthetic pathways (Eudes et al., 2017). In that study syringyl lignin and both methanol soluble tricin and lignin-bound tricin were reduced in a brown midrib sorghum with a mutated SbCOMT gene. Here ROMT9/OsCOMT was more highly expressed in the rice pericarp samples than the embryo samples of both cultivars, likely reflecting the higher expected lignin content of the pericarp samples.

The rice and sorghum examples of reduced, but not eliminated, levels of tricin in the ROMT/COMT knockout lines indicates the existence of additional *O*-methyltransferases that are involved in tricin biosynthesis (Lam, Liu & Lo, 2015; Eudes et al., 2017). Likely candidates are two *O*-methyltransferases designated ROMT15 and ROMT17. ROMT15 and ROMT17 were active against several flavonoids, including the flavonol tricetin, and both methylated the 3′ and 5′ hydroxyls of tricetin, producing tricin (Lee et al., 2008). The ROMT15 expression pattern was similar to that of ROMT9/COMT in that it was more highly expressed in the pericarp samples than the embryo samples. ROMT17 was more highly expressed in the IAC600 embryo than the IAC600 pericarp but higher in the Cocodrie pericarp than the Cocodrie embyo. The expression pattern of ROMT15 suggests it is a likely candidate for involvement in the biosynthesis of tricin.

Overall, the reason for the lack of detectable tricin in the Cocodrie pericarp samples is not clear from the gene expression levels of the enzymes required for tricin biosynthesis. The tricin precursor chrysoeriol was detected in all the pericarp samples. The gene encoding the enzyme involved in the conversion of chrysoeriol to selgin, CYP74B4, was expressed at a higher level in the Cocodrie pericarp than in the IAC600 pericarp where free tricin was detected. Also the genes for the possible *O*-methyltransferase enzymes, ROMT15 and ROMT17, possibly involved in the conversion of selgin to tricin were expressed at higher or similar levels in the Cocodrie pericarp as compared with the IAC600 pericarp. Possibly any tricin produced in the Cocodrie pericarp was bound to lignin and would not be detected in our samples.

### Expression of anthocyanin biosynthetic genes

The purple pericarp color of IAC600 is due to the presence of anthocyanins, primarily cyanadin-3-*O*-glucoside. The structural genes of the anthocyanin pathway in rice are known (Fig. 1) (Shih et al., 2008; Oikawa et al., 2015). Their expression was highly up-regulated in the IAC600 pericarp relative to the other samples (Table 4). The log2 fold changes of the genes required for anthocyanin biosynthesis, chalcone synthase, flavanone 3-hydroxylase, dihydroflavonol-4-reductase, and anthocyanin synthase I between the IAC600 pericarp and the Cocodrie pericarp samples ranged from 8.7 to 10. Chalcone isomerase was not as highly up-regulated as the other anthocyanin biosynthetic genes. Similar levels of up-regulation of these genes in black rice cultivars, as determined by real time PCR methods, were reported previously (Oikawa et al., 2015). Activation of the anthocyanin genes in the pericarp of pigmented cultivars is due to ectopic expression of the bHLH transcription factor *Kala4* (Os04g0557500), which has an 11 kb insertion in the promoter region relative to non-pigmented cultivars (Oikawa et al., 2015). *Kala4* was overexpressed in the IAC600 pericarp relative to the other samples (Table 4).

The flavonol quercetin is formed by the desaturation of dihydroquercetin, also a precursor in the anthocyanin pathway, catalyzed by flavonol synthase. Quercetin-3-*O*-glucoside was detected only in the IAC600 pericarp samples. Quercetin-3-*O*-glucoside has previously been reported in some purple rice genotypes (Kim et al., 2010; Sriseadka, Wongpornchi & Kayanakorn, 2012; Poulev et al., 2018). Flavonol synthase enzymes have been characterized in several species (Cheng et al., 2014), although none have yet been functionally characterized in rice. However, anthocyanin synthase I, which is highly expressed in the IAC600 pericarp, has been shown to also act on dihydroquercetin in addition to leucocyanidin in vitro and in planta (Reddy et al., 2007). They proposed that anthocyanin synthase I substitutes for flavonol synthase in rice. The conversion of dihydroquercetin to quercetin by anthocyanin synthase I would be consistent with quercetin-3-*O*-glucoside being detected only in the IAC600 pericarp tissue.

## Discussion

Here, we report that the higher levels of tricin previously detected in the bran of the purple pericarp cultivar IAC600 relative to the light brown pericarp cultivar Cocodrie (Poulev et al., 2018) is a result of synthesis of tricin in both the pericarp and embryo of IAC600 but only in the embryo of Cocodrie. The higher levels of tricin in the bran of several purple pericarp rice cultivars in a screen of rice cultivars of varying pericarp color (Poulev et al., 2018) suggests that synthesis of tricin in the pericarp as well as in the embryo may be common in purple pericarp rice. Only one previous study has examined the location of flavonoids in rice grain. In the cultivar Nipponbare, tricin, and chrysoeriol and their conjugates were found exclusively in the embryo fraction; none were detected in the pericarp (Galland et al., 2014). Here the tricin precursor chrysoeriol was detected in the pericarp samples of both IAC600 and Cocodrie indicating that the flavone pathway up to chrysoeriol is functioning in the Cocodrie pericarp. The genes required for the conversion of chrysoeriol to tricin were expressed in the Cocodrie pericarp samples, so it may be that any tricin synthesized in the Cocodrie pericarp was lignin-bound and would not have been extracted in our samples. How the partitioning of tricin between binding to lignin and remaining as free tricin is regulated is not known. The higher levels of free tricin previously detected in the bran of IAC600 (Poulev et al., 2018) may be due to the apparent overall higher synthesis of tricin in the IAC600 pericarp such that there is free tricin remaining after a subpopulation becoming bound to lignin. Future research could examine the levels of lignin-bound tricin in developing rice pericarp tissue.

The early steps in the flavonoid biosynthetic pathway, chalcone synthase and chalcone isomerase, are shared between the anthocyanin and flavone pathways and these genes were highly up-regulated in the IAC600 pericarp. The expression of the flavone-specific pathway genes was considerably lower in all the samples relative to the level of the anthocyanin pathway genes in the IAC600 pericarp. In rice regulation of expression of the flavone-specific pathway genes appears to be independent from that of the anthocyanin pathway genes as it is in maize (Morohashi et al., 2012). Future research could address the factors regulating the biosynthesis of flavones in developing rice grains. The detection of free tricin in the pericarp of IAC600 and not in Cocodrie does not seem to be due to the high expression levels of chalcone synthase and chalcone isomerase in the IAC600 pericarp generating more precursors for the flavone biosynthetic pathway since, on a ng per gram basis, the levels of free tricin in the pericarp and embryo samples of IAC600 were similar. If the synthesis of free tricin in the pericarp of IAC600 is a general feature of purple pericarp rice cultivars, there may be another shared feature of the cultivars independent of the synthesis of anthocyanins.

Higher levels of tricin in whole grain rice could increase its nutritional value. A transgenic approach was used to produce flavonoids, including tricin, in rice endosperm by heterologous expression of flavonoid biosynthetic genes whose expression was driven by endosperm specific promoters (Ogo et al., 2013). Such an approach would increase the nutritional value of polished rice. However, whole grain rice has many nutrients in addition to flavonoids. Brown pericarp rice is more widely cultivated than purple pericarp rice due to the generally lower yields of purple pericarp rice cultivars (Ji et al., 2012). Rahman, Lee & Kang (2015) proposed that the reduced yields of purple pericarp rice may be due to anthocyanin interference in chlorophyll synthesis in the pericarp leading to reduced photosynthesis. Increasing tricin in brown rice is an attractive approach to improving the nutritional quality of rice. Since the regulation of the flavone specific pathway genes appears independent of the regulation of the anthocyanin pathway genes, it may be possible to increase the tricin levels in brown rice by crossing high tricin purple pericarp cultivars with brown pericarp cultivars and selecting for brown pericarp progeny with higher levels of tricin in the grain. Zarei et al. (2018) have also proposed incorporating the goal of increasing bran nutritional components into rice breeding efforts. Improving the nutritional value of the bran fraction of rice and encouraging consumer acceptance of whole grain rice could contribute to improving human nutrition (Dipti et al., 2012).

In contrast to the prospect of increasing levels of tricin in rice grain for improved human nutrition, decreasing tricin biosynthesis was proposed as a target for manipulation in efforts to improve cell wall digestibility in plants grown for biomass. A rice flavone synthase II (CYP93G1) tDNA insertion mutant was deficient in tricin, and had altered lignin composition and increased biomass digestibility (Lam et al., 2017).

The RNA-Seq data generated in this study was analyzed for expression of flavonoid genes. In the future it will also be a good resource for analysis of genes involved in rice pericarp and embryo development and for cultivar variation in gene expression of developing grains.

## Conclusions

Tricin is a methylated flavone that has been reported to have health benefits. The only dietary sources of tricin are whole grains, where tricin is found in the bran fraction. Rice bran from different cultivars has been found to have a wide range of tricin levels, with the purple pericarp cultivars having the highest levels. Here, we analyzed tricin content in developing embryos and pericarp tissue from a purple pericarp cultivar and a light brown pericarp cultivar. The difference in total tricin level between the cultivars was largely due to the presence of tricin in both the embryo and pericarp of the purple pericarp cultivar, but only in the embryo of the light brown pericarp cultivar. Overall, most of the tricin in the purple pericarp cultivar was in the pericarp tissue. Better understanding of the regulation of tricin biosynthesis in pericarp tissue may lead to approaches to increase tricin in the more widely cultivated light brown and brown pericarp cultivars of rice.

## Additional Information and Declarations

### DNA Deposition

The following information was supplied regarding the deposition of DNA sequences: The sequence data is available at NCBI under the BioProject ID PRJNA490738.

### Data Availability

The following information was supplied regarding data availability: The raw data is included in the article.
